# A critical review on applications of the Avrami equation beyond materials science

**DOI:** 10.1098/rsif.2023.0242

**Published:** 2023-06-21

**Authors:** Kiana Shirzad, Christopher Viney

**Affiliations:** ^1^ Graduate Program in Materials and Biomaterials Science and Engineering, University of California, Merced, CA 95343, USA; ^2^ Department of Materials Science and Engineering, University of California, Merced, 95343, CA, USA

**Keywords:** Avrami equation, COVID-19, epidemiology, nucleation and growth, phase transformations, SIR (susceptible-infected-removed) model

## Abstract

The Johnson–Mehl–Avrami–Kolmogorov (JMAK) formalization, often referred to as the Avrami equation, was originally developed to describe the progress of phase transformations in material systems. Many other transformations in the life, physical and social sciences follow a similar pattern of nucleation and growth. The Avrami equation has been applied widely to modelling such phenomena, including COVID-19, regardless of whether they have a formal thermodynamic basis. We present here an analytical overview of such applications of the Avrami equation outside its conventional use, emphasizing examples from the life sciences. We discuss the similarities that at least partially justify the extended application of the model to such cases. We point out the limitations of such adoption; some are inherent to the model itself, and some are associated with the extended contexts. We also propose a reasoned justification for why the model performs well in many of these non-thermodynamic applications, even when some of its fundamental assumptions are not satisfied. In particular, we explore connections between the relatively accessible verbal and mathematical language of everyday nucleation- and growth-based phase transformations, represented by the Avrami equation, and the more challenging language of the classic SIR (susceptible-infected-removed) model in epidemiology.

## 1. Introduction

The Avrami equation was originally developed, and is now widely used, to model the progress of phase transformations in materials systems. It is a simple sigmoidal function

1.1 $$f(t)=1-\exp(-kt^{n})\;,$$

where f(t) is the fraction of material that is transformed as a function of time, and *k* and *n* are constants that are extracted from the model [1]. It originated in classical nucleation theory (CNT) and represents the convergence of analyses performed independently by Johnson & Mehl [2], Avrami [3–5], and Kolmogorov [6]; see [7] for detailed chronology and access to appendices from the original paper by Johnson and Mehl. It is sometimes formally referred to in the literature as the JMAK equation (or the KJMA equation, depending on the conventions of the authors who are referencing the equation).

The Avrami equation has been used to describe the progress of thermodynamic phase transformation of materials in both the physical sciences (for example precipitation [8] and deformation-induced martensitic transformation [9] in metal alloys, crystallization in amorphous polymers [10] and metallic glasses [11], and self-assembly of supramolecular structures [12]) and the life sciences (for example crystallization of fats [13], proteins [14], haem [15] and bone minerals [16]). Beyond the context of materials phase transformation, there are many other examples of changes that initiate and spread through processes similar to crystal nucleation and growth. There have been previous efforts to apply the Avrami equation to the kinetics of many such non-thermodynamic transformations. The focus of this review is to critically explore the extent of common ground between various processes approximated by the Avrami model, and thus to assess the validity and limitations of these adaptations of the model. In particular, we consider the justifiability of using an Avrami model to describe the progression of disease in a susceptible population.

## 2. Background: the Avrami equation and thermodynamic phase transformations

### 2.1. Classical nucleation theory

The process of materials phase transformation is often divided into two stages: nucleation and growth. Nucleation is the beginning of formation of a new thermodynamic phase with lower free energy, in a parent phase with higher free energy [17]. Nucleation can be instantaneous (time-independent, occurring at the same time for all transforming regions in a system) or sporadic (occurring at different times for different transforming regions).

Nucleation occurs spontaneously via random fluctuations in the local arrangement of atoms (or molecules) in the parent phase [18] in two ways: *homogeneous* nucleation, when free-standing nuclei form without aids from external nucleation sites, and *heterogeneous* nucleation, which is aided by crystal defects or foreign surfaces [17,19].

Among several theories that have been developed for understanding the nucleation and growth of a new thermodynamic phase, CNT is the most common [17]. Assuming homogeneous nucleation of spherical solid nuclei with radius *r* in a liquid parent phase, there are two components to the Gibbs free energy of nucleation: the volume free energy that works towards formation of stable nuclei, and the surface energy that works against it. (For nucleation in a solid matrix, there is a third energy component; it manifests as a strain, caused by the density difference between the product and the parent phase, and works against nucleation.) Therefore, the change in Gibbs free energy is

2.1 $$\Delta G=\frac{4}{3}\pi r^{3}\Delta G_{v}+4\pi r^{2}\gamma \;=\;\mathrm{volume}\;\mathrm{effect}\;+\;\mathrm{surface}\;\mathrm{effect}.$$

**Figure 1.**
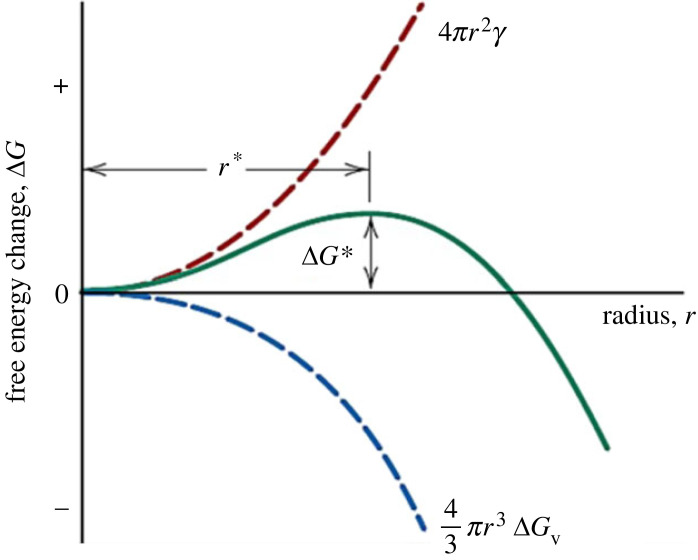
Schematic plot of free energy versus nucleus radius (adapted from Callister & Rethwisch [19]).

The volume free energy change of phase transformation, $\Delta G_{v}$, is always negative; the interfacial energy, γ, between regions of transformed and parent material is always positive. Since smaller nuclei have a higher surface-to-volume ratio, their surface energy cost is greater than the released volume free energy. For larger nuclei, the volume free energy dominates, making the total Gibbs free energy negative; therefore, the nuclei are viable and can continue to grow and become ‘grains’. This competition is shown in figure 1 (adapted from Callister & Rethwisch [19], Chapter 10).

Differentiation of equation (2.1) with respect to *r* gives the critical radius of stable nuclei, $r^{*}$*,* as

2.2 $$r^{*}=-\frac{2\gamma }{\Delta G_{v}}\;.$$

For heterogeneous nucleation, the surface energy term contains the collective effect of the solid–liquid, solid–impurity, and liquid–impurity interfacial energies.

CNT is developed based on the following assumptions: [17,20–23]

(1) Nuclei have the same properties (e.g. density, structure and composition) as the transformed phase.
(2) Nuclei are spherical and have a sharp boundary with the parent phase; the boundary thickness is negligible compared with the size of the nuclei.
(3) Capillary approximation: the surface tension (energy per unit area, γ) of nuclei is assumed to be similar to that of a flat surface, i.e. independent of nucleus curvature, roughness and size.
(4) One-step nucleation: cluster formation and internal organization into the new phase happen simultaneously, without temporary formation of any intermediate or metastable state.

There are many transformations where the observed behaviour departs from these assumptions. There are cases where the nuclei do not have the same density or structure as the transformed phase [24], the interface with each nucleation cluster is an ordered region with a thickness that is comparable to the cluster radius [25], small nuclei have highly curved surfaces that disobey capillary approximation [23,26], or critical nuclei form by aggregation of intermediate disordered clusters of particles followed by later appearance of periodic crystal structure [24].

Once the overall free energy is negative, the now-stable nuclei can enter the second stage of transformation; growth.

### 2.2. Kinetics of transformation and growth

The time dependence of transformation has been described by the Avrami equation [1], noted above as equation (1.1). Cantor [27] dedicated a full chapter to the Avrami equation in his book ‘The Equations of Materials' where he selects and expounds on the most important equations in materials science.

**Figure 2.**
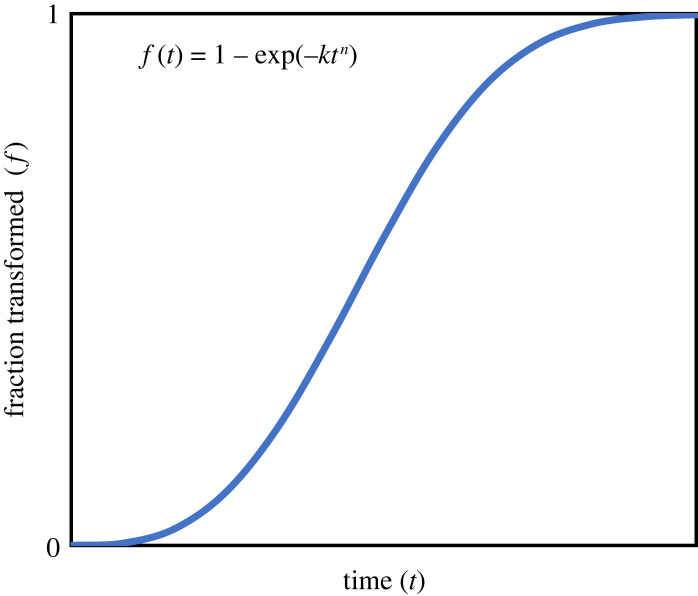
Typical plot of progress of a phase transformation at constant temperature.

The equation creates a sigmoidal curve (figure 2). Initially, transformation is slow; this stage is followed by a period of rapid grain growth due to the increasing availability of interfacial area for atoms or molecules to attach to. Eventually the transformation slows towards completion, because the process will run out of transformable material and growing grains will impinge on each other. This impingement can be in the direct form of physical contact between two (or more) growing grains. It can also occur at a distance when the diffusion fields of growing precipitate grains overlap, i.e. the grains compete for the same compositional resources [28].

The constants *k* and *n* in the Avrami equation are system specific. Avrami [4] uses different notations for these parameters; our convention here is consistent with recent literature. The Avrami coefficient *k* is a function of both nucleation rate and growth rate, and is related to the geometry of the growing nuclei [29]. The Avrami exponent *n* depends on dimensionality of growth, *d*, and on whether nucleation is instantaneous or sporadic [29]. We can write *n* = *n*_N_ + *n*_G_, where *n*_N_ and *n*_G_ embed the nucleation and the growth components respectively [27]. If nucleation is instantaneous—such as in heterogeneous nucleation with a fixed number of nucleation sites—then *n*_N_ = 0, while for continuous or sporadic nucleation *n*_N_ = 1. Some studies [11,30] report nucleation rates that change over time. Accordingly, the value of the term $n_{N}$ can have a value that is neither 0 or 1 (table 1). The growth component depends on the growth mechanism and will be addressed in more depth in the following section.

**Table 1.** Values of $n_{N}$ for different cases of nucleation rate*.*

$n_{N}=1$	constant sporadic nucleation rate
$n_{N}=0$	instantaneous nucleation
$n_{N}>1$	nucleation rate increases with time
$0<n_{N}<1$	nucleation rate decreases with time

### 2.3. Growth mechanisms and Avrami exponent

Phase transformations can be categorized based on the mechanisms involved in growth: (i) interface migration, associated with atoms or molecules locally rearranging from the structure of the parent phase into that of the product phase; (ii) diffusion in a multi-component system, causing changes in chemical composition [31]. A transformation can involve one or both of these mechanisms. In the latter case the slower mechanism controls the overall rate of the transformation.

#### 2.3.1. Interface-controlled growth

**Figure 3.**
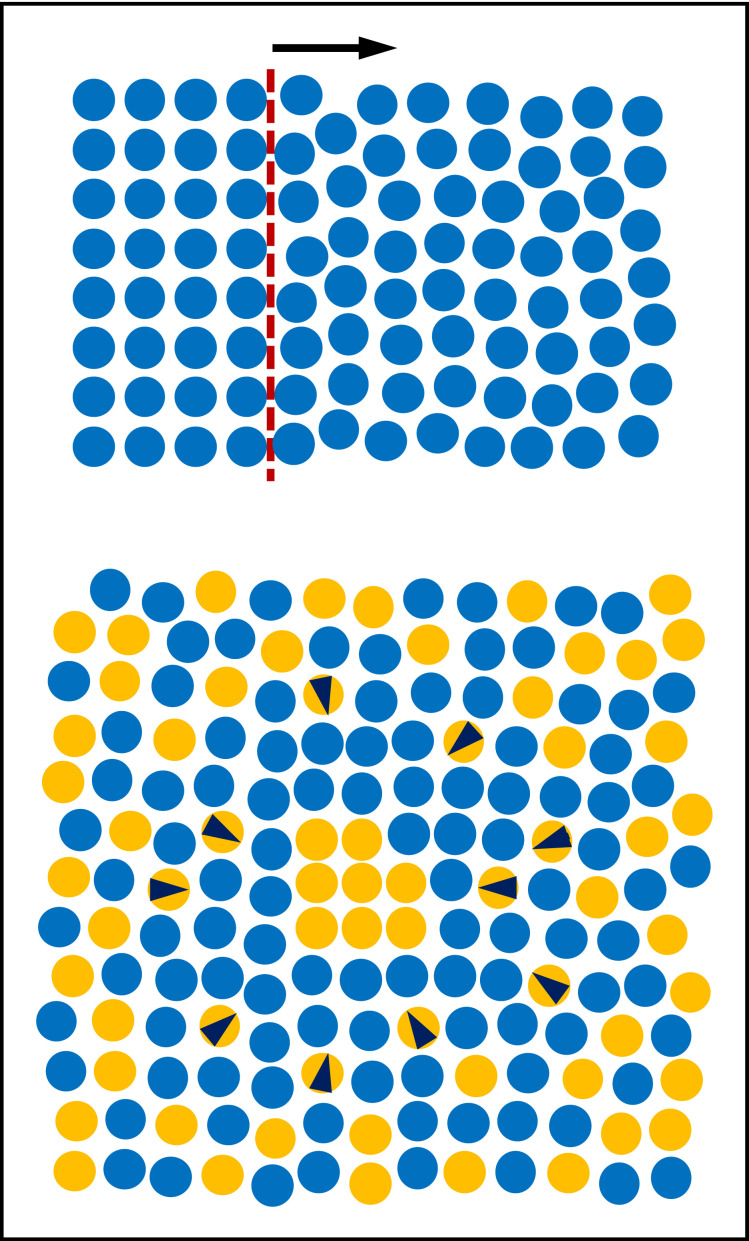
Schematic representations of interface-controlled growth (top) and diffusion-controlled growth (bottom).

This mechanism involves processes that take place in close proximity to the interface. There is negligible change in volume and no change in composition as the interface moves (figure 3, top). This type of growth is linear with time [28]. Examples include polymorphic phase changes, solidification and solid-state structural changes in single component materials, and recrystallization [28,32].

Interface-controlled growth depends only on the attachment of the atoms or molecules from the parent phase onto the new phase at the interface. Since the growth of a grain in each dimension is linear in time (*r* ∝ *t*), the time-dependence of volume for a three-dimensional grain is *v*(*t*) ∝ *t*^3^. For sporadic nucleation (*n*_N_ = 1), the value of the Avrami exponent is *n* = 1 + 3 = 4 for spherical or three-dimensional growth, *n* = 1 + 2 = 3 for plate-like or two-dimensional growth and *n* = 1 + 1 = 2 for needle-like or one-dimensional growth. For instantaneous nucleation, the time dependence of nucleation is zero (*n*_N_ = 0); therefore, the Avrami exponent will be *n* = 3, 2 and 1 for spherical, plate-like and needle-like growth respectively [27]. When grains impinge on each other, growth stops in that direction. This termination of growth is referred to as ‘hard impingement’ [28].

Constant growth rate is a valid assumption for interface migration into a matrix of constant composition; the growth rate is independent of the position of the interface and, as a result, independent of time. However, when a new phase with a different composition (and possibly structure) precipitates out of the matrix, constant growth rate cannot be assumed, because the availability of precipitating atoms around the interface decreases over time [27].

#### 2.3.2. Diffusion-controlled growth

When the matrix around the growing precipitate is depleted of solute atoms (figure 3, bottom), continued growth will depend on the diffusion of solute atoms through the matrix at ever greater distances from the interface. In this mechanism, contrasting with the original assumptions of Avrami [3–5], growth in each dimension is proportional to the square root of time [28]. Examples of diffusion-controlled growth include precipitate formation in the Al–Cu alloys [33] used in aircraft construction and the precipitation of intermetallic compounds in magnesium alloys [34].

Since each growing dimension of a grain follows the relationship *r* ∝ *t*^1/2^, the time-dependence of volume for a three-dimensional grain is now *v*(*t*) ∝ *t*^3/2^. For sporadic nucleation (*n*_N_ = 1), the value of the Avrami exponent is *n* = 1 + 3/2 = 2.5 for three-dimensional growth, *n* = 1 + 2/2 = 2 for two-dimensional growth and *n* = 1 + 1/2 = 1.5 for one-dimensional growth. For instantaneous nucleation (*n_N_* = 0), the Avrami exponent is *n* = 1.5, 1 and 0.5 for spherical, plate-like and needle-like growth respectively [27].

For diffusion-controlled growth, the diffusion fields of growing grains start to overlap before the grains physically touch, and the grains will have to compete for the available solute in the matrix. This competition results in a gradual decrease in growth rate until growth stops. This phenomenon is called ‘soft impingement’ [28].

## 3. Applications of the Avrami equation in the life sciences and beyond

Though the JMAK model was developed to describe the progress of phase transformation in a material system, its application has not been limited to materials science. The model has been used in other fields of research, including life sciences examples such as oncology [35–40], ecology [41–45] and epidemiology [46–50]. Some systems from those contexts can be regarded as undergoing a ‘phase transformation’, and non-thermodynamic driving forces analogous to undercooling and supersaturation can be identified. An Avrami coefficient (*k*) and an Avrami exponent (*n*) can be extracted from data for the system being modelled, and can be interpreted on the basis of assumptions that are often justifiable. Dimensions in these cases are any settings through which the transformation propagates, rather than merely the three-dimensional space coordinates. The energy cost of the transformation can be compared with any factor that works against the transformation, similar to the surface energy of the interface in materials phase transformations.

### 3.1. Applications in genetics studies

Li *et al*. [51] studied the transfer of antibiotic resistant genes in bacteria. They developed an approximation formula, based on an adaptation of the Avrami equation by Avramov [46] for modelling the spread of an epidemic, and found outstanding agreement (*R*^2^ = 0.996) between experimental results and the model. Their use of this equation is merely data fitting, and is not explained in the context of phase transformations.

O'Malley *et al*. [52] applied the Avrami equation to describe the spread of invasive gene clusters. Mutations are described as nucleating randomly and independently in space and time, creating clusters of invasive alleles. Clusters that are larger than the critical size will continue to grow until they reach a sufficiently large radius, and the radial velocity approaches an asymptotic value. The authors appear to use the concepts of limiting radius and asymptotic (finite) growth velocity interchangeably as a means of incorporating impingement into their model.

Richards *et al*. [53] modelled the viability decline that seed ageing causes in gene banks. They do not provide any comparison between phase transformation and loss of gene viability, and their choice of Avrami exponent (*n* = 2) is made without invoking any explicit analogue of Avrami growth kinetics.

Ralph & Coop [54,55] persuasively applied the Avrami model to the geographical spread of selected alleles in a human population that is adapting to a global selection pressure. An advantageous allele, that can arise and spread locally, will spread until it encounters (impinges on) an allele with the same advantage that is spreading through the species from a different location.

Herrick *et al*. [56] adopted an Avrami model to study the kinetics of DNA replication. They justify this adoption via the following parallels: (i) random activation of DNA replication origins (*nucleation*), (ii) synthesis of the replication structure (*growth*), and (iii) coalescence of two replicated DNA domains once they touch one another (*impingement*). Assuming a constant growth rate, they describe the kinetics of DNA replication similarly to one-dimensional crystal growth. Their modified Avrami model fits the experimental data from DNA replication in *Xenopus*, yields valid description of the time dependence of replication initiation, provides the initiation rate of new replication origins and can be generalized to other organisms. Jun & Bechhoefer [57,58] also studied the application of a one-dimensional Avrami model to DNA replication. Their implementation accounts for small, broken-up DNA fragments, which do not require the assumption of infinitely long DNA strands made by Herrick *et al*. [56].

### 3.2. Applications in cancer studies

González *et al*. [35] discovered diffusion-controlled growth and soft impingement processes during tumour growth. They used a modified Avrami model to describe the tumour growth, noting that, as in crystal growth, tumour growth is initially exponential and then slows as the tumour asymptotically approaches its final volume, resulting in a sigmoidal curve. Villar Goris *et al*. [36] compared the Avrami model and the Gompertz model (a sigmoidal function widely used in biology to describe growth of various living organisms or tissues [37]) in predicting growth kinetics of tumours; they achieved similar results from the two models. In this case, the Avrami coefficient is related to the tumour growth rate, and the progress of the transformation is measured in terms of the addition of cancer cells to the solid tumour.

Dobrzyński *et al*. [38] modelled the dose dependence of radiation-induced cancer, describing this dependence as a sigmoidal function. They consider continuous irradiation of cells at fixed dose rates, and focus on the dose dependence evolution of cancer cells, neglecting the natural time-dependent evolution. The Avrami model is shown to be more universal in fitting the data, compared with a multi-stage model. The exponent *n* = 3.68 is considered close to 4 and interpreted as voluminous growth, implying (but not stated) that nucleation is sporadic. Dobrzyński *et al*. [39] also used the Avrami equation to model the probability of cancer transformation as a function of the number of irradiation-induced mutations in a cell. The implications of the resulting Avrami exponent (*n* = 2) are not addressed.

Fornalski & Dobrzyński [40] employ the Avrami model to describe the cancer transformation probability for a single cell. They treat each oncogene as a cancer cluster on the DNA chain. They interpret the Avrami coefficient in terms of the distribution of mutations necessary for cancer transformation, and the Avrami exponent in terms of the transformation dynamics.

### 3.3. Applications in biochemical processes

Cope [59,60] presents thermodynamic and kinetic arguments that sigmoidal time-dependent data from diverse biological phenomena can be thought of as phase transitions. He applies the Avrami equation to these systems, generating the Avrami exponent values *n* listed in table 2. Cope highlights that the values of *n* are not distributed randomly; they cluster around values that have specific interpretations in phase transformation. Also, physiologically and/or biochemically related processes exhibit similar *n* values. For example, *n* ≈ 2.0 for cases of growth in animal and plant species, and 1.0 < *n* < 1.3 for light-generating processes. Szasz *et al*. [61] provide mathematical insights regarding why the Avrami equation is so broadly applicable to the diverse systems addressed by Cope.

**Table 2.** Avrami exponent for different biological processes [60]. Notice that the *n* values for growth in plants and animals cluster around 2 (green shading) and the values for light-related processes cluster between 1 and 1.3 (blue shading).

biological phenomenon	*n*
growth of bacteria	4
growth (length) of regenerating leg of salamander	2.3
synthesis of chlorophyll in maize plant	2.2
growth (weight) of rat	2.0
growth (height) of sunflower plant	1.9
K^+^ conductance decay in nerve axon	1.9
K^+^ leakage from poisoned muscle	1.7
dried green leaf IR phosphorescence decay	1.28
myosin splitting of ATP	1.24
muscle tension during tetanic contraction	1.21
cytochrome c IR phosphorescence decay	1.16
firefly flashlight decay	1.16
melanin IR phosphorescence decay	1.08
fresh green leaf IR phosphorescence decay	1.00

Karšaj & Humphrey [62] modelled the production of fibrin from fibrinogen in intraluminal thrombus. They present references to existing, more complex models for the underlying kinetics, and argue that the Avrami model is much simpler and has the same efficacy in describing the data.

### 3.4. Applications in ecology

Kuśnierz & Łomotowski [41] adapted the Avrami equation to model growth in an algal suspension. Nucleation, growth and stagnation of algal agglomerates follow the curve of the Avrami model. The authors consider the random spatial distribution of individual algal cells, and the effect of their division and contacts on overall growth rate. Tran *et al*. [42] modelled the biofouling of mortar by green algae, using the Avrami equation, and assuming random nucleation and a constant growth rate of the clusters. The surface fraction that is colonized by algae follows a sigmoidal curve. Garbowski *et al*. [43] modelled the volume fraction of algal particles having a given diameter in wastewater. The constant *k* encompasses the rate of nucleation and growth of the particles, and *n* is related to the geometry of particles. While the values of *k* and *n* are not reported, the authors state that *n* can be between 1 and 4, aligned with Avrami's principles. The fitted Avrami equation exhibits great agreement with their data (*R*^2^ = 0.99).

Graziani & Quagliarini [44], and Quagliarini *et al*. [45], modelled algal growth on sandstone, limestone and clay bricks. They set *n* = 4 (assuming that nucleation and growth are always sporadic and three-dimensional, respectively), and they fit data by adjusting *k* (which they recognize as depending on the nucleation rate of new particles and their growth rate). By this means, they obtain a close match between model and data; *R*^2^ = 0.978 or higher [44]. The authors conclude that the model can predict the algal growth [45]; however, they have used the Avrami equation solely to fit their experimental data. Also, they invoke considerations external to the Avrami model to explain why *k* values vary by an order of magnitude for data obtained from different samples of the same material, and across different materials.

Company *et al*. [63] modelled the loss of seed viability of native grass species in soil. Their interpretation of the Avrami coefficient and exponent is limited to how they describe the shape of the curve. Korniss & Caraco [64] used the Avrami model to describe the time-dependence of density of a resident plant species upon invasion by a superior species. They assume that homogeneous nucleation and growth of clusters of the invasive species in a *d*-dimensional volume cause a decay in the resident species. Nucleation is translated to successful introduction of the invading species; a cluster of the invasive species will spread if it is larger than a critical size.

Sangwal [65] applied the Avrami model (which he calls the Avrami-Weibull function) to germination of tomato seeds in soil—and to growth of bacteria in cheese, milk and broth. His results show, without explanation, that the Avrami model fits the data better than other (Verhulst, Gompertz) growth models.

### 3.5. Applications in epidemiology

Avramov [46] proposes that spread of disease (and many other types of disturbance, in diverse systems) can be modelled as diffusion-based processes, and therefore by the Avrami equation. He borrows language from the SIR epidemiology model [66] (where *S* denotes the susceptible state, *I* denotes the infective state, and *R* denotes the removed or recovered state) to categorize the total population into three groups. Individuals in a total population are considered as connected cells (locally or long-distance) in a *d*-dimensional lattice. Individuals (cells) in *I* state transfer the infection to the neighbouring cells in *S* state. In comparison with phase transformation, Avramov assumes that *S* state corresponds to the untransformed phase, and *I* and *R* states together account for the transformed phase. The rate of long-distance transfer corresponds to the rate of formation of critical-sized nuclei, and infection spreads locally only one unit in space per unit of time (i.e. growth rate is constant). The Avrami exponent is *n* = *d* + 1, therefore assuming sporadic nucleation, and the Avrami coefficient is related to the combined long-distance and local propagation rates. Avramov notes that *d* must be determined experimentally and is not simply correspondent to the geographical area through which the disturbance is spreading.

Driven by the 2019 coronavirus disease (COVID-19) pandemic, there have been several preprint articles on the application of the Avrami equation in spread of infection.

Augusto *et al*. [47] used nucleation theory and the Avrami equation to model the fraction of population infected by COVID-19 in Brazil. They (tenuously) assume temperature to be equivalent to the ‘degree’ of people who are on the streets spreading the pandemic at the borders of contaminated neighbourhoods, without long-distance transmission. Their Avrami coefficient represents the constant expansion rate of COVID-19 cases over time, and their Avrami exponent is the ability to form new infection nuclei and is related to long-distance transmission (e.g. by air travel). Each infected individual is assumed to create one infection cluster (nucleus) when entering a previously uncontaminated region. Based on their results, *n* = 3.8 and *n* = 5.7 give the closest fits to the data from two Brazilian states. The authors do not provide any interpretation of these values.

Takase [48] investigated the spread of COVID-19 in Japan, with an approach derived from previous work on a ferroelectric polarization reversal phenomenon in polymers [67] where the dipole switching was modelled by the Avrami equation. He states, without elaboration, that semicrystalline polymers resemble the complexity of human societies, and that the time dependence of COVID-19 spread has similar characteristics to those of polarization reversal. Both one-dimensional and two-dimensional growth models show good agreement with the epidemic data in Japan. Takase suggests that the spread of the epidemic in Japan was rate-limited by three factors: ‘the initial susceptible, the domain growth rate, and the nucleation decay constant’. No rationale is presented on how the assumptions and attributes of the Avrami model translate into the context of epidemiology.

Tonchev [49] fitted the Avrami model to mortality data from COVID-19 in four European countries, obtaining *n* = 3.4 for Spain, *n* = 4.8 for the United Kingdom, *n* = 7.2 for France and *n* = 3.4 for Italy. He does not provide reasoning for this application of the Avrami equation. While the model fits the sampled data (up to 27 April 2020), it severely underestimates the cumulative number of deaths and when it would plateau in all the four countries (errors vary from around 65% to around 100% according to the real COVID-19 data [68]).

Vilone & Andrighetto [50] used the Avrami equation to include the time-dependence of the infection rate in modelling the infected population during an epidemic's initial period. While they introduce the Avrami equation as a phase transition model, they seem to suggest this model merely as a mathematical tool, without any parameter interpretation in epidemiology.

### 3.6. Applications in chemistry

The Avrami equation is widely used in solid-state reaction kinetics (e.g. redox reactions [69–71] and hydrolysis reactions [72]). In these contexts, it is sometimes referred to as the Avrami-Erofeev equation [73–75].

Kosaka *et al*. [69] and Lorente *et al*. [70] studied the kinetics of iron oxide redox reaction, where *k* is the reaction rate constant and *n* represents the dimensionality of nucleation and growth. Their graphs show a good match between experimental and estimated results. Eigen *et al*. [71] modelled redox reactions in Fe_2_NiO_4_/yttrium stabilized zirconia composites using two Avrami equations in series, each corresponding to one of the two steps of the overall oxidation process. They justify their range of *n* (0.80 < *n* < 2.25) with an initial instantaneous nucleation at locations where the redox mass is available to the gas phase, followed by a diffusion-controlled transport of iron to gas/oxide interfaces. Alasmar *et al*. [72] studied the hydrolysis of Nd-Ni-Mg compounds. Their estimated *n* = 1.17 is interpreted as being consistent with a surface-controlled reaction.

Mubarak *et al*. [76] applied the Avrami model to the adsorption of heavy metal ions from groundwater onto zeolite nanocomposites, and achieved close fits to the experimental results (minimum *R*^2^ = 0.96). No interpretation is provided for the resulting Avrami exponent, which is smaller than 0.5 for all their cases.

### 3.7. Applications in social studies

Ausloos & Petroni [77] used the Avrami equation to study the dynamics of religions and the number of adherents. They consider religion like any other social variable which can develop, spread and adapt in a society, and can be studied from a macroscopic (i.e. number of religions) or a microscopic (e.g. number of adherents) viewpoint. Their model was applied to religions in Mexico by González-Aviles & Campuzano [78], who defined nucleation as the initial change in the state of a small but stable religion and approximated the percentage of adherents (with respect to the world population) as a function of time. The Avrami exponent (−0.502 ≤ *n* ≤ 1.379) was introduced as a measure of the increase or decline in the percentage of adherents; however, the accompanying explanation appears to be inconsistent with the numerical interpretation of *n* in the context of crystal growth.

Sangwal [65] applied the Avrami equation to the citation of papers written by selected top-cited authors. He compares the cognitive pressure on authors (the driving force to cite a paper) to the thermodynamic free energy threshold of phase transformation. The total cognitive citation pressure is related to the ‘attractiveness’ of the paper that is being cited. The excellent fit of the Avrami model to citation data (*R*^2^ > 0.99) is interpreted in terms of diffusion and integration (equivalent to atoms being incorporated into a growing crystal) of published knowledge. However, there is no attempt to include (and there may be no room for) the concept of impingement in the analogy.

### 3.8. Application in product market analysis

Marianna See [79] used the Avrami equation to model the market fate of steel and aluminium. This application refers to three stages of material production and marketing: (i) introduction of the material and gradual discovery of methods of development or manufacturing; (ii) market acceptance and increased production rate due to increased demand; (iii) market saturation and eventual decrease in production due to depleted resources and decreased demand. These stages are compared with the Avrami curve: initial slow growth, rapid exponential growth, and impingement. The author states that the Avrami parameter *k* is equivalent to the sum of coefficients representing innovation (an external influence) and imitation (an internal influence), and that *n* is equivalent to the ratio of the internal and external influences. The correspondence to the original meanings of *k* and *n* is not discussed.

### 3.9. Applications in failure analysis

Ohring & Kasprzak [80] discuss the applicability of the Avrami model in failure analysis. In an initially defect-free volume, seeds of damage (e.g. voids or precipitates) germinate at a constant nucleation rate. Nuclei are assumed to be spherical, and to grow at a constant rate until impingement occurs. The volume fraction of damaged material can be expressed by the Avrami equation where the coefficient *k* embeds temperature, nucleation rate and growth rate, and *n* = 4 as required by the above assumptions.

Rodbell *et al*. [81] applied the Avrami equation to elucidate the failure mechanism caused by electromigration in metallic thin-film interconnects. Electromigration of metal ions is related to the nucleation, growth and coalescence of voids, caused by a supersaturation of vacancies. The driving force for electromigration is provided by both temperature and applied electromagnetic field. Numerical values of *n* in this study are consistent with the classical geometric interpretation of nucleation and growth.

## 4. Justifications for applying the Avrami model to describe change in a system

### 4.1. Fundamental assumptions in Avrami's original development of the theory

Avrami [3–5] describes phase transformations based on the following assumptions and principles that are broadly supported by experiment:

(1) The phase transformation is initiated by germ nuclei (i.e. smaller than the critical size) that pre-exist in the parent phase. After the critical size is exceeded, nuclei can grow steadily and become *grains* in a process called ‘*granulation*’. The number of germ nuclei per unit nucleation space depends on the degree and duration of supercooling.
(2) The number of germ nuclei per unit region decreases from the initial number in two ways: (i) they become growth nuclei (i.e. larger than the critical size) due to temperature- and composition-dependent free energy fluctuations; (ii) they are swallowed by growing grains of the new phase whose growth rate is also a function of temperature and concentration.
(3) There is an ‘isokinetic range’ of temperatures in which the nucleation and growth rates are proportional to each other, i.e. they have the same temperature variation, and the kinetics of phase transformation can therefore be described by the model even in non-isothermal conditions [28]. (The existence of this isokinetic range is not a requirement of the Avrami model, but an observation.)
(4) Growth rate *G* of all grains is constant. This means that the germ nuclei quickly go from the initial slow growth to the constant growth rate, so that any incubation period of growth nuclei is neglected.
(5) Growth stops where hard impingement happens (but continues elsewhere until the entire system has transformed).
(6) The grains that could have formed if their nuclei had not coincided with an already-occupied area, or had not been consumed by the growing grains, are called ‘*phantom grains*’. The total volume of the grains, including the phantom grains, is called ‘*extended volume*’. The actual transformed volume is the fraction of the extended volume that excludes the phantom grains. The concept of extended volume in the derivation of equation (1.1) is independent of any assumption about isothermal or isokinetic conditions [4,82,83].

Application of the Avrami equation to a system may be possible if the system matches this framework. Polymer crystallization often violates assumptions of constant nucleation rate and constant growth rate (because the motion of the polymer chains is constrained), and 100% crystallinity is unattainable in practice (due to impurities, polydispersity and entanglements) [84,85]. Consequently, although the Avrami equation has been used in polymer crystallization [86], it usually works only for the early stages and the data deviate from the model as the transformation progresses [85]. A similar limitation pertains to the later stages of crystallization in thin samples of metallic glass, where the Avrami exponent changes during the course of the transformation [87].

The fact that the Avrami equation can usefully describe diverse life science phenomena, but can fail in describing some materials crystallization data, establishes that a ‘thermodynamic phase transformation’ is neither necessary nor sufficient for applying the Avrami model. From a statistical viewpoint, many transformations can be described by this model, even if they do not follow its dimensional and geometrical conventions. In a general sense of CNT, viable nuclei of change can form and grow if they overcome a nucleation barrier. Replacing the Gibbs free energy (a function of the geometry of the transformed regions; equation (2.1)) with *θ* (a function of an undefined variable x>0), we can write

4.1 $$\theta (x)=\;Bx^{b}+Ax^{a},$$

where *B* represents the agencies benefitting (in favour of) the change and *A* represents the agencies against the change, both of which are assumed to have a power law relationship with *x* as a generalization that maintains accordance with CNT. Both *b* and *a* are real numbers and b>a. The critical point of θ(x) yields the critical $x^{*}$ value

4.2 $$\frac{d\theta (x)}{dx}=\;Bbx^{b-1}+Aax^{a-1}=0\;\;\;\to \;\;\;x^{*}=\sqrt[{b-a}]{\frac{-Aa}{Bb}}\;.$$

It is easily shown that (dθ(x)/dx)>0 for any $x<x^{*}$, and (dθ(x)/dx)<0 for any $x>x^{*}$. Since *b* and *a* are not confined to integers, we can argue that this nucleation barrier can exist in a non-Euclidean space, where growth can occur in non-Euclidean dimensions. This argument helps us to address the concept of ‘dimensions’ where *growth* is not happening in a conventional geometrical space, such as in spread of invasive alleles in a species or spread of infection through a human population. Other constraints may also be revised. For example, modelling shows that the final volume fraction of precipitates does not significantly depend on whether nucleation rate is constant or decreasing [88]. Also, assumption of complete transformation can be circumvented by replacing the ‘total volume’ with ‘total transformable volume’ or ‘equilibrium transformed volume’ [89].

### 4.2. Generalization of Avrami kinetics

As a prelude to modelling any non-thermodynamic transformation (e.g. due to cancer, infection or a trend) by the Avrami equation, it is helpful to find defensible equivalences that can justify such application, and thereby differentiate modelling from mere curve fitting. This key support for the analogy is often missing, which detracts from possible interpretation of the constants *k* and *n*, even in cases where the equation can be made to fit the data. This issue has been discussed previously [90] regarding application of the Avrami model in absorption processes [91]. The same issue is apparent in some epidemiology literature where the Avrami equation is used simply as a sigmoidal fit to COVID-19 pandemic data, without justification of the model parameters [47–50].

Extending the principles of CNT and the Avrami equation to the broader context of initiation and spread of a ‘change’, we identify a *driver* equivalent to Δ*G*_v_ and a *resistance* equivalent to *γ.* For example, in the case of a behavioural change, Δ*G_v_* may be related to intrinsic incentives, and *γ* may depend on cultural norms opposing that behaviour. Given that many of the reviewed cases are not rooted in thermodynamics, we will not attempt to define analogues of additional thermodynamic parameters such as response functions, thermodynamic potentials or order parameters.

We should recognize that interpretation of the Avrami parameters in any context may depend on how the modelled ‘change’ is measured. This consideration is prompted by polymer crystallization data where the value of the Avrami exponent depends on whether the crystallized fraction is measured directly or indirectly [92].

Epidemiology offers a potentially rich—and relatively unexplored—context for the Avrami equation to describe the progress of the infected (transformed) fraction of population. Nearly one-third of the global disease burden is associated with infectious diseases [93]. COVID-19 infected over 80 million people in 2020 alone [68]. Disease outbreaks can cause long-lasting social and economic damage. Factors driving an increasing probability of infection outbreaks include population density, antibiotic resistance, air travel and environmental changes [94–96]. Implementing the concept of transformation and the principles of the Avrami equation in this context can help to create a broadly accessible cross-disciplinary language for communicating the growth of an epidemic. However, a more formal justification for applying the Avrami equation in epidemiology is first needed. It is essential to understand both the Avrami theory and the epidemiological parameters that determine the fate of an infectious disease, and to draw logical parallel between the two systems. In the next section, we provide a description of one of the most commonly used models in epidemiology, the SIR model.

## 5. Modelling epidemics, and the comparison with phase transformation

### 5.1. SIR epidemiology model

The SIR (susceptible-infected-removed) model is a compartmental epidemiology model, originating in the work of Hamer [97], Ross [98], Kermack and McKendrick [99] and others [100,101]. In this model, the total population, *N*, is divided into three subpopulations: *susceptible* group, S(t); *infected* group who can spread the disease to the susceptible individuals, I(t); and *removed* population who have recovered or died from the disease, R(t). The model comprises three differential equations which provide a mathematical basis to describe spread of a disease

5.1 $$\frac{dS}{dt}=\;-\frac{\beta IS}{N},\;\;\;\frac{dI}{dt}=\frac{\beta IS}{N}-\gamma I,\;\;\;\frac{dR}{dt}=\;\gamma I.$$

*S*, *I* and *R* are functions of time. β is the constant infection rate, and is the product of the average number of exposures per unit time (*α*) and the probability of infection during an exposure (*μ*) [102]. γ is the constant recovery rate or removal rate of the infected individuals; it is related to the reciprocal of the infectious period, which is the average number of infectious days before transitioning from the *I* state into the *R* state [99,103]. The ratio of β to γ is the basic reproductive number, $R_{0}$, defined as ‘the average number of secondary cases that one case would produce in an entirely susceptible population’ [104]. The effective reproductive number, $R_{e}$, is similarly defined but in a population made up of both susceptible and infected individuals [105]

5.2 $$R_{0}=\frac{\beta }{\gamma },\;R_{e}=\frac{\beta }{\gamma }\frac{S(t)}{N}.$$

$R_{0}$ quantifies the initial contagiousness of the infection, while $R_{e}$ is a time-dependent value. $R_{e}$ serves as a threshold value, determining whether the infection will decline and die out $\left(R_{e}<1\right)$ or whether it will prevail and turn into an epidemic $\left(R_{e}>1\right)$ [100,105,106].

The SIR model is based on the following assumptions [66,99]: (i) the total population (S+I+R) is large and constant; (ii) the outbreak is a short-term situation; (iii) birth of new individuals is neglected, as well as the death from causes other than the infection; (iv) no incubation period is considered for the infection, so individuals become infectious immediately after getting infected; (v) the subpopulations are closed compartments, so transformation to the *R* state is irreversible and reinfection is not possible; (vi) individuals in the subpopulations are randomly and homogeneously distributed (the probability of contact between all members is equal).

### 5.2. Reasoned extension of the Avrami model to epidemiology

We now compare the Avrami and SIR models: drawing parallels based on the main elements of the models that represent and simplify the real phenomena, and noting the relative accessibility of the Avrami framework as a communication tool. The comparisons start with nucleation and continue to growth and impingement, and are summarized in table 3.

(1) Phase transformation is assumed to happen in an infinite geometrical space (JMAK) [107]. Most epidemiological models (including SIR [66]) assume a very large total population.
(2) Based on CNT, there is a threshold radius $r^{*}$ after which the nuclei are stable and phase transformation is energetically favourable and can proceed (JMAK) [19]. In epidemiology, there is a threshold value for the persistence of the epidemic, which is the reproduction number of a virus, $R_{0}$, (SIR) [105]. It should be noted that the threshold $R_{0}$ value is by definition a global threshold for the continuation of the epidemic and not for the formation of infected clusters. However, $r^{*}$ is local and clusters smaller than this critical value will disappear and cannot initiate formation of the new phase.
(3) CNT treats the germ nuclei in the same way as growing grains: the size dependence of properties such as surface energy is neglected (JMAK) [108]. Similarly, epidemiological models generally consider homogeneous population characteristics, neglecting individual differences such as personal contact networks (SIR) [109].
(4) Nuclei are randomly distributed within the parent phase (JMAK) [17]. Infected individuals are randomly distributed in the population (SIR) [66].
(5) Germ nuclei quickly transition from the initial slow growth to the constant growth rate and any incubation period of growth nuclei is neglected (JMAK) [4]. Infected individuals immediately become infectious, without any incubation exposed state (SIR) [66].
(6) All grains in a given system maintain their geometry during growth (JMAK) [4]. Infection spreads around all infected individuals in a consistent pattern due to the random and equal contact probabilities between all members of the population (SIR) [66].
(7) Grain growth is hindered by impingement (JMAK) [4]. Spread of infection around an infected person will be restricted if they encounter other infected people (SIR) [66].
(8) Both models contain case-specific rates that embody the drive for initiation and progress of the transformation. In phase transformations, the Avrami coefficient (*k*) encompasses nucleation rate and growth rate (and therefore temperature) (JMAK) [110]. In epidemiology, the reproduction rate $\left(R_{0}\right)$ embeds the infection rate and transmissibility of the disease (SIR) [102]. More precisely:
  (8.1) Infection rate (β) parallels the rate of diffusion and attachment of new molecules or atoms to growing grains.
  (8.2) The average number of contacts per unit time (*α*) affects the probability of the spread of infection; similar to how diffusion coefficient affects grain growth in material systems.
(9) Supercooling drives the initiation of a new phase in materials (JMAK) [29]. Transmissibility or infectiousness of a pathogen (*µ*) causes new infections (SIR) [102,111]. The role of supercooling in materials can be performed by other drivers, such as evaporation-induced supersaturation in a solution [112,113].
(10) CNT assumes that the nuclei of the new phase form through a single step process (JMAK) [17]. Transition from susceptible state to infected occurs in a single step: contracting the disease (SIR) [66]. Although there may be many differences between the parent and transformed phases, it is possible to distinguish between them on the basis of a single parameter, e.g. density in the case of phase transformation and infection in epidemiology.

**Table 3.** Summary of the parallels between the Avrami and SIR models.

characteristic	JMAK (Avrami)	SIR
1. no space limits	phase transformation in an infinite space	infection spreading in a very large total population
2. existence of a threshold	nuclei are viable if their radius is larger than the critical radius	infection sustains an epidemic if *R_0_* > 1
3. holistic scaling	nuclei have the same properties as the growing grains	individuals have the same contacts and immunity as the population average
4. random initiation points	random distribution of nuclei	random distribution of susceptible and infected
5. no incubation time	quick transition of nuclei from slow growth to constant growth rate	immediate transition of individuals from infected to infectious
6. isotropic propagation	similar growth geometries for all particles	similar spreading patterns around each infected cluster
7. termination process	hindered growth when transformed regions meet	hindered spread when infected individuals meet
8. constant reaction rates	constant rate of diffusion and attachment to the growing body	constant possibility of spread per unit time
9. presence of a driver	degree of supercooling drives transformation	transmissibility of pathogen drives spread
10. single-step process	one-step transformation; can describe in terms of a single parameter	one-step transition from susceptible to infected state

We recognize that there are also several differences between the two systems, including the possibility of long-range transmission of disease via air travel and cruise ships, while growth of a new thermodynamic phase occurs at the interface with the parent phase. Also, straightforward crystallization and the Avrami model do not include an equivalent of the recovered group that appears in the SIR model. However, there is value in appreciating the similarities that can support the use of the Avrami equation as a simple mathematical tool with only two parameters to model the evolution of a pandemic, as well as many other life sciences phenomena.

Interestingly, the idea of harnessing a kinetic perspective to describe the propagation of disease was suggested in 1925 by McKendrick [114], pre-dating even his seminal publication [99] that laid the groundwork for the SIR model. Defining rate coefficients β and γ then provided the means for developing a kinetics-based approach to describing epidemics. These coefficients each relate to single processes. By contrast, the kinetic parameters *k* and *n* that describe phase transformations both embed the time dependence of two processes—nucleation and growth. Thus, there is no direct correspondence between the main parameters of the Avrami and SIR frameworks.

### 5.3. Extending the context of the SIR model

Finally, we note that the SIR model has itself been adapted to unconventional contexts, to model transformations as diverse as the spread of song popularity [115], political influence [116], rumors [117] and gun ownership [118]. While we have focused on life sciences applications of the Avrami equation, the spirit of interdisciplinary science invites us to also consider whether the SIR model might have relevance in describing materials phase transformations. For example, many phase transformations proceed via a kinetically necessary intermediate state [17,119]; compartments of susceptible, infected and recovered could have analogues in the parent, intermediate and final transformed phases.

## 6. Conclusion and outlook

The Johnson–Mehl–Avrami–Kolmogorov (Avrami) model, widely used in crystal nucleation and growth studies, offers a simple method for describing and/or predicting the time dependence of a variable that measures the spread of change in many different circumstances. Applications extend across several non-thermodynamic contexts that intersect the life sciences, including genetics, oncology, biochemistry, ecology and epidemiology. We have attempted to give an account of the justifications for, and limitations of, such generalization. It is apparent that contextual validation often remains incomplete, and may even be unattainable in every detail. While the numerical values of the model parameters, *k* and *n*, may serve to characterize a transformation, interpretation of the underlying reasons for those values should be executed with caution.

The COVID-19 pandemic has highlighted a need to explain the sigmoidal profile of disease propagation effectively, to broad and often sceptical audiences, so that timely mitigation can be adopted to ‘flatten the curve’. The relative simplicity of the Avrami equation, and the accessible language of everyday transformations such as solidification of liquid water into ice, provide a potentially useful communication framework. We hope that this review will promote relevant interdisciplinary conversation.

## Data Availability

This article has no additional data.
